# Water-Soluble, Biocompatible Polyphosphazenes with Controllable and pH-Promoted Degradation Behavior

**DOI:** 10.1002/pola.27002

**Published:** 2013-11-22

**Authors:** Sandra Wilfert, Aitziber Iturmendi, Wolfgang Schoefberger, Kushtrim Kryeziu, Petra Heffeter, Walter Berger, Oliver Brüggemann, Ian Teasdale

**Affiliations:** 1 Institute of Polymer Chemistry, Johannes Kepler University LinzWelser Street 42, 4060, Leonding, Austria; 2 Institute of Organic Chemistry, Johannes Kepler University LinzAltenberger Street 69, 4040, Linz, Austria; 3 Faculty of Science, University of South BohemiaBranišovská 31, 370 05, České Budějovice, Czech Republic; 4 Institute of Cancer Research and Comprehensive Cancer Center of the Medical University of Vienna, Medical University of ViennaBorschkegasse 8a, 1090, Vienna, Austria; 5 Research and Platform “Translational Cancer Therapy Research,”Vienna, Austria

**Keywords:** biodegradable polymers, biocompatible polymers, polymer therapeutics, polyphosphazenes, water-soluble polymers

## Abstract

The synthesis of a series of novel, water-soluble poly(organophosphazenes) prepared via living cationic polymerization is presented. The degradation profiles of the polyphosphazenes prepared are analyzed by GPC, ^31^P NMR spectroscopy, and UV–Vis spectroscopy in aqueous media and show tunable degradation rates ranging from days to months, adjusted by subtle changes to the chemical structure of the polyphosphazene. Furthermore, it is observed that these polymers demonstrate a pH-promoted hydrolytic degradation behavior, with a remarkably faster rate of degradation at lower pH values. These degradable, water soluble polymers with controlled molecular weights and structures could be of significant interest for use in aqueous biomedical applications, such as polymer therapeutics, in which biological clearance is a requirement and in this context cell viability tests are described which show the non-toxic nature of the polymers as well as their degradation intermediates and products. © 2013 The Authors Journal of Polymer Science Part A: Polymer Chemistry Published by Wiley Periodicals, Inc. J. Polym. Sci., Part A: Polym. Chem. **2014**, *52*, 287–294

## Introduction

Functional polymers with a biodegradable backbone, biocompatible degradation products, and controlled degradation profiles are of great promise for biomedical applications.^1^–^3^ Although providing eminent water-solubility and biocompatibility, the well-studied polyethylene glycol (PEG)^4^,^5^ and poly(*N*-(2-hydroxypropyl)methacrylamide (HPMA)^6^,^7^ lack in degradability, thus leading to accumulation in the body when used as high molecular weight polymers, limiting their applicability to some extent.^8^,^9^ In addition to polyacetals and polyketals,^10^ polyglutamic acid,^11^ dendritic polyesters,^12^ and recent work about polyphosphoesters,^13^ polyphosphazenes certainly represent a promising biodegradable class of polymers for biomedical applications.^14^,^15^ Because of their unique, degradable backbone, the properties of polyphosphazenes greatly depend on the choice of organic substituents attached to the phosphorus atoms.^16^ The major precursor polymer, poly(dichlorophosphazene), can be obtained by thermal ring opening polymerization or living cationic polymerization, followed by macromolecular substitution of the chlorine atoms by a wide range of organic nucleophiles such as amines and alkoxides.^17^ Substitution of the polyphosphazene chain with suitable nucleophiles allows the design of poly(organophosphazenes) with versatile properties,^17^ multifunctionality,^18^ and tunable hydrolytic sensitivity,^19^ all of which represent crucial features for polymers used in biomedical applications.

The incorporation of various specific substituents remarkably affects the hydrolytic degradability of the polymer.^20^ In this context, substituents such as amino acid ester,^1^,^21^–^24^ imidazolyl,^25^ glucosyl,^26^ glyceryl,^27^ and *N*-ethylpyrrolidone^28^ yield hydrolytically sensitive polyphosphazenes with different degradation rates. The hydrolytic stability can be fine-tuned by adapting the hydrophilicity/hydrophobicity of the substituent,^25^ as well as mixed-substitutent polyphosphazenes^29^,^30^ with the amount of side groups, that generate hydrolytic sensitivity adjusted. There are several different pathways suggested for the degradation process of polyphosphazenes including the hydrolysis and thus release of the substituted side groups, followed by the attack of water on the phosphazene backbone, leading to the formation of hydroxyphosphazenes and phosphazanes.^21^ These intermediates then undergo further chain cleavage, generating a pH buffered system of nontoxic phosphate and ammonia.^31^

The purpose of this study was to synthesize a series of water-soluble polyphosphazenes via the living cationic polymerization^32^,^33^ with biocompatibility and controlled biodegradability. It is well-established that the rate of degradation of polyphosphazenes can be tailored through the addition of amino acid ester side groups.^3^,^21^ However, amino acid esters generally produce hydrophobic water insoluble polyphosphazenes, ideal for tissue engineering^34^ or implantable biomaterials,^35^ but not suitable for aqueous applications. Thus, with the aim to extend their applicability to aqueous applications, we sought to prepare water-soluble variants with similarly good control of degradability. Although a mixed substitution with both amino acid esters and hydrophilic groups is also possible,^14^ this route clearly has its upper incorporation limits (and thereby degradation limit), with increasing hydrophobic portion also leading to amphiphilic polymers and furthermore aggregation.^36^ The methods described herein show how this can be achieved through the direct incorporation of the prepared pegylated amino acid linkers, significantly broadening the spectrum of water soluble degradable polyphosphazenes achievable, with degradation rates ranging from several days to months. Furthermore, we describe how decreases in pH-value lead to rapid acceleration of the degradation profile as well as preliminary cell viability tests showing the biocompatibility of the polymers and the benign nature of their degradation products.

## Experimental

### Materials and Methods

All synthetic procedures were performed air-free either in a glovebox (MBRAUN) under argon or under nitrogen using standard Schlenk line techniques. The glassware was dried in an oven at 120 °C prior to use. The amino capped statistical poly(ethylene oxide-*co*-propylene oxide), sold under the trade name Jeffamine M-1000 (referred to herein as M-1000) with a nominal molecular weight of 1000 g mol^−1^, was donated by Huntsman Performance Products (The Netherlands) and used as received. Solvents were dried using standard laboratory methods. PCl_5_ was purified by sublimation and stored in the glovebox under argon. NEt_3_ was dried over molecular sieves and distilled prior to use. All other chemicals were purchased from Sigma–Aldrich and used without further purification.

^1^H NMR spectra were measured on a Bruker Avance 300 spectrometer and a Bruker DRX 500 spectrometer using CDCl_3_ or D_2_O as an internal reference. The ^31^P NMR experiments were conducted on both magnets at resonance frequencies of 121 and 202 MHz, respectively, using 85% phosphoric acid as an external standard. Gel permeation chromatography (GPC) was performed on a Viscothek GPCmax instrument using a PFG column from PSS (Mainz, Germany) (300 mm × 8 mm, 5 μm particle size). The samples were eluted with DMF containing 5 mM LiBr as the mobile phase at a flow rate of 0.75 mL min^−1^ at 60 °C. The molecular weights were calculated relative to polystyrene standards from PSS using a conventional calibration of the refractive index detector. Dynamic light scattering (DLS) was performed on a Malvern Zetasizer Nano ZS instrument with a detection angle of 173° and a 4 mW He-Ne laser operating at a wavelength of 633 nm. The samples were filtered through a nylon microfilter (0.2 μm) prior to the measurement. The hydrodynamic diameter of the polymers in deionized H_2_O (1 mg mL^−1^) at 25 °C was determined from the volume size distribution and is expressed as a mean value. FTIR spectra were obtained with a Perkin Elmer Spectrum 100 FTIR spectrometer equipped with an ATR accessory. UV–Vis spectra were performed on a Perkin Elmer Lambda 25 UV/VIS spectrophotometer.

### Synthetic Procedures

#### Trichlorophosphoranimine (Cl_3_P=N-SiMe_3_)

The monomer was prepared using a method adapted from the literature.^37^,^38^ LiN(SiMe_3_)_2_ (24.22 g) (145 mmol) was dissolved in 400 mL anhydrous diethyl ether. The reaction was cooled to 0 °C and stirred for 30 min. PCl_3_ (12.66 mL) (19.87 g, 145 mmol) was added dropwise at 0 °C and the solution was stirred for 30 min. SO_2_Cl_2_ (11.70 mL) (19.53 g, 145 mmol) was added and the mixture was stirred for another hour at 0 °C. The reaction was then filtered through celite and the volatiles were removed under vacuum. The product was purified twice by vacuum distillation (50 °C, 4 mbar) to yield Cl_3_P=N-SiMe_3_ as a colorless liquid which was stored under an inert argon atmosphere at −35 °C. Yield 15.00 g (46%).

^1^H NMR (300 MHz, CDCl_3_, δ): 0.17 (s, 9H) ppm. ^31^P NMR (121 MHz, CDCl_3_, δ): −54.37 ppm.

#### Synthesis of Poly(dichlorophosphazene)

The polymers were synthesized according to the procedure for the living cationic polymerization of trichlorophosphoranimine.^32^ In the glovebox, the monomer Cl_3_P=N-SiMe_3_ (0.45 g, 2.01 mmol) and the initiator PCl_5_ (0.02 g, 0.08 mmol) were dissolved in CH_2_Cl_2_ and stirred at room temperature. After 12 h, the solvent was removed under vacuum. The obtained poly(dichlorophosphazene) was used for macromolecular substitution without further purification. Yield quantitative.

^31^P NMR (121 MHz, CDCl_3_, δ): −18.16 ppm.

#### M-1000-Gylcine and M-1000-Valine

The following representative procedure describes the coupling reactions of M-1000 and the BOC-protected amino acids Boc-Gly-OH and Boc-Val-OH, respectively. Boc-Val-OH (1.63 g, 7.50 mmol), *N*-hydroxysuccinimide (0.86 g, 7.50 mmol), and *N*,*N*′-dimethylaminopyridine (0.09 g, 7.50 mmol) were dissolved in 80 mL CH_2_Cl_2_ and cooled to 0 °C. In a second flask, 1.55 g *N*,*N*′-dicylcohexylcarbodiimide (7.50 mmol) was dissolved in 15 mL CH_2_Cl_2_ and transferred to the reaction mixture at 0 °C. The mixture was allowed to warm to room temperature and stirred overnight. The formed precipitate was then removed by filtration. The filtrate was added to a solution of 7.50 g M-1000 (7.50 mmol) in CH_2_Cl_2_ and stirred for 2 days. The reaction was extracted twice with 10% ammonium chloride, twice with 5% sodium hydrogen carbonate, saturated sodium chloride, and dried over MgSO_4_. The solvent was removed under vacuum and the product further dried under high vacuum to yield M-1000-Valine-Boc as a white wax-like product. Yield 6.39 g (71%).

^1^H NMR (300 MHz, CDCl_3_, δ): δ 1.07 (m, 8H), 1.37 (s, 9H), 3.30 (s, 3H), 3.57 (m, 87H) ppm. The M-1000-Valine-Boc was deprotected in TFA/CH_2_Cl_2_ (1/3) for 3 h. The solvent was removed under vacuum, redissolved in CH_2_Cl_2_ followed by washing the product with 5% sodium hydrogen carbonate and saturated sodium chloride. The organic phase was dried over MgSO_4_ and removed under vacuum. The product was further dried by co-evaporation with toluene and chloroform to obtain M-1000-Valine. Yield: quantitative.

#### Synthesis of Polymers

**Scheme 1:**
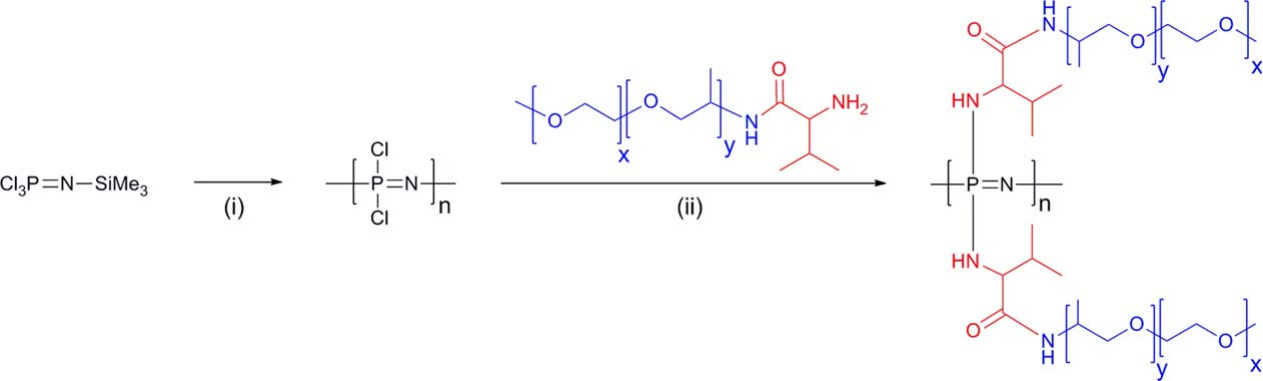
Synthesis of polymer 3 via the living polymerization of trichlorophosphoranimine followed by macromolecular substitution. Reagents and conditions: (i) PCl_5_, CH_2_Cl_2_, room temperature, 12 h and (ii) NEt_3_, THF, room temperature, 24 h. [Color figure can be viewed in the online issue, which is available at http://wileyonlinelibrary.com.]

The following typical example procedure describes the synthesis of polymer **3** (Scheme 1). In the glovebox, poly(dichlorophosphazene) (0.15 g, 0.67 mmol, 1 eq) was dissolved in anhydrous THF. A solution of M-1000-Valine (1.76 g, 1.60 mmol, 2.4 eq) in THF and NEt_3_ (0.16 g, 0.22 mL, 1.60 mmol, 2.4 eq) was then added to the polymer solution and stirred for 24 h at room temperature. The suspension was filtered to remove the formed ammonium chloride and the solvent was concentrated under vacuum. The polymer was purified by several precipitations into chilled diethyl ether from THF and dried under high vacuum. Yield 0.57 g (38%).

FTIR (solid): ν_max_ = 2883 (C=H), 1653 (C=O), 1109 (C=O) cm^−1^, ^1^H NMR (500 MHz, D_2_O, δ): 0.99 (br, 9H), 3.21 (s, 3H), 3.53 (s, 82H) ppm. ^31^P NMR (202 MHz, D_2_O, δ): 1.05 ppm.

All other polymers were synthesized accordingly, with the same molar ratio of monomer to initiator (25/1) and thus with a theoretical number of repeat units *n* = 50, and the amount of side groups adjusted to obtain the desired polymers. For polymers **2** and **4** with two different side groups, the two substituents were mixed in a molar ratio of 1/1 in THF followed by the addition of the polymer precursor and NEt_3_. All polymers were dried under vacuum to give colorless viscous to wax-like products in yields of 30–70% (see Supporting Information for further characterization data).

### Degradation Studies

The degradation behavior of the polymers was studied by GPC, ^31^P NMR, and UV–Vis spectroscopy. For ^31^P NMR degradation studies, 20 mg of the polymer was dissolved in 0.5 mL D_2_O (pH 7) and D_2_O acidified with HCl (pH 2, enhanced degradation conditions), respectively. The samples were incubated in the denoted solvents at room temperature and the changes of the phosphorus signals were monitored over a time period of up to 2 months.

The polymers were incubated in TRIS buffer (pH 7.4), sodium acetate buffer (pH 5), or acidified H_2_O (pH 2, enhanced degradation conditions) in a concentration of 4 mg mL^−1^ at 37 °C during the time of analysis. The polymer degradation medium was tested for the presence of inorganic phosphate. Aliquots of the degradation medium were taken in regular time intervals and mixed with a reagent solution consisting of ammonium molybdate, ascorbic acid, sulfuric acid, and potassium antimonyl tartrate (method adapted from the literature).^23^ UV–Vis analysis of the mixtures was performed after 15 min of incubation time at 885 nm. The concentration of phosphate was calculated from a calibration curve using potassium dihydrogen phosphate and is given as the percentage compared to the theoretical phosphate amount that can be released from the polymer backbone. For further testing, samples of 0.75 mL were taken and the water was removed followed by GPC analysis as described above.

### Cell Viability Studies

#### Cell Culture

The following human cell lines were used in this study: the colon carcinoma cell line HCT116 (a gift from Dr. Vogelstein, John Hopkins University, Baltimore, MD), the ovarian carcinoma cell line A2780 (from Sigma–Aldrich) and the hepatoma model Hep3B (from American Type Culture Collection, Rockville, MD). All cells were grown in a humidified atmosphere with 5% CO_2_ at 37 °C in RMPI 1640 medium supplemented with 10% fetal bovine serum (FCS), with the exception of HCT116 cells which were cultivated in McCoy's culture medium with 10% FCS. Cultures were regularly checked for Mycoplasma contamination.

#### Cytotoxicity Assays

Cells were plated (2 × 103 cells/well) in 100 μL cell culture medium per well in 96-well plates. After a recovery period of 24 h, the polymers dissolved in 100 μL growth medium (0.01–10 μM) were added and the cells exposed for 72 h (pH 7.4). Polymer stock solutions were either freshly prepared or stored for 4 or 8 weeks at 37 °C. The proportion of viable cells was determined by MTT assay following the manufacturer's recommendations (EZ4U, Biomedica, Vienna, Austria). Given values are normalized to control samples without any treatment. For experiments at pH 6.0, 2-(*N*-morpholino)ethanesulfonic acid (MES)-buffered medium was used for polymer incubation.

## Results and Discussion

### Synthesis of Polymers

**Scheme 2:**
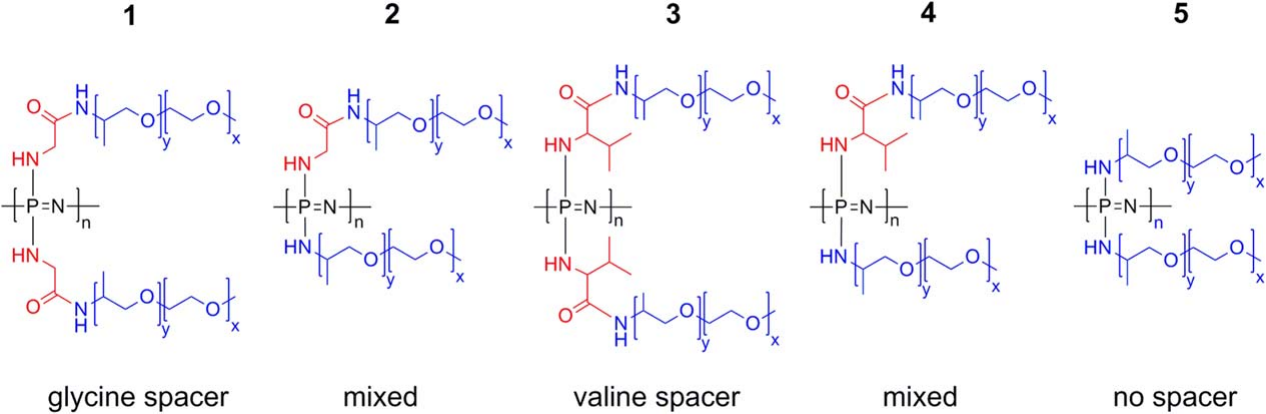
Structures of hydrophilic graft poly(organophosphazenes) 1–5. N.B. the structures shown for polymers 2 and 4 are simplified, with the macromolecular substitution resulting in a random, mixed substitution pattern. [Color figure can be viewed in the online issue, which is available at http://wileyonlinelibrary.com.]

Poly(dichlorophosphazenes) were synthesized via the room temperature living cationic polymerization of trichlorophosphoranimine. The chlorine atoms of the polymeric precursor were then replaced with the highly water-soluble oligomer Jeffamine M-1000, an amine-capped polyether belonging to the Jeffamine family, which interestingly have been reported to have better cell uptake than PEG.^39^ The amino acid spacers, glycine and valine, were incorporated between the polyphosphazene backbone and the hydrophilic side chains to give a series of graft poly(organophosphazenes) (Scheme 2) with varied hydrolytic stabilities. ^31^P NMR spectra of the polymers **1**–**5** showed single, broadened peaks in the range from 0 to 4 ppm indicating the absence of chlorine atoms, at least up to NMR detection limits. A large excess of amine and long reaction times were used to ensure complete removal of the chlorine atoms since remaining P-Cl units would be expected to significantly enhance the rate of degradation.^31^ As the stable polymer **5** (no amino acid spacer) was shown to be reproducibly stable under neutral conditions, it is presumed that consistent complete replacement of the chlorine atoms (see degradation studies below) was achieved under the conditions applied. The polymeric structures and purity of the polymers were also confirmed by ^1^H NMR and ATR-FTIR spectroscopy (see Supporting Information).

The living polymerization provides a route to polyphosphazenes with molecular weights that can be controlled by the initial ratio of monomer to initiator, but is not able to prepare polyphosphazenes with large chain lengths (above *n* = 75) with good reproducibility.^32^,^40^ Through the attachment of larger organic components, higher molecular weights are accessible, in this case the substitution with M-1000 side groups, to yield polyphosphazenes with theoretical molecular weights over 100 kg mol^−1^ (*n* = 50) (Table1). The apparent molecular weights *M*_n_(exp) measured by GPC were estimated using a conventional calibration versus linear polystyrene standards and were a factor of 5–10 lower than the calculated values *M*_n_(theo), due to the highly branched nature of the polymers. However, the relatively narrow molecular weight distributions *M*_w_/*M*_n_ in the range of 1.1–1.3 clearly demonstrate the living and controlled nature of the polymerization procedure, especially considering that the attached side chains are not monodisperse. Furthermore, the hydrodynamic diameters of the polymers in aqueous environment were measured by DLS and lie in the range of 5.9–7.0 nm.

**Table 1:** Characterization of Polymers 1–5

Polymer	R_1_	R_2_^a^	*M*_n_ (theo)^b^ (kg mol^−1^)	*M*_n_ (exp)^c^ (kg mol^−1^)	*M*_w_ /*M*_n_	*d*^d^ (nm)
**1**	M-1000-Gly	–	108	20	1.16	6.5 (±0.2)
**2**	M-1000-Gly	M-1000	105	11	1.34	6.8 (±0.1)
**3**	M-1000-Val	–	112	13	1.34	7.0 (±0.1)
**4**	M-1000-Val	M-1000	107	15	1.20	6.7 (±0.1)
**5**	M-1000	–	102	14	1.23	5.9 (±0.1)

a Molar feed ratio of R_1_/R_2_ was 1/1.

b Calculated from the initial molar ratio of monomer to initiator (25/1, *n* = 50).

c Apparent value determined by GPC with conventional calibration versus linear polystyrene standards.

d Measured by DLS in deionized H_2_O.

### Degradation Studies

**Figure 1:**
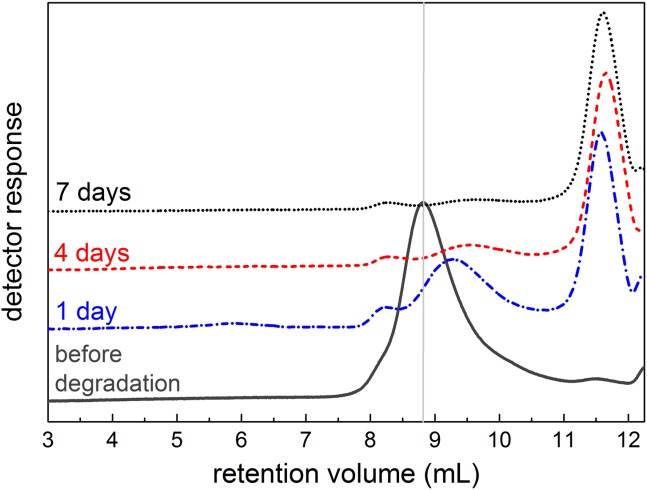
Degradation studies of polymer 3 with GPC under accelerated conditions in acidified H_2_O (pH 2) over 7 days. The arising peak at around 11.6 mL (ca. 1000 g mol^−1^) confirms the hydrolysis and thus release of the side groups. [Color figure can be viewed in the online issue, which is available at http://wileyonlinelibrary.com.]

The hydrolytic degradation and decline of molecular weight of the synthesized polymers were investigated under enhanced degradation conditions in water at pH 2 using gel permeation chromatography. As an example, GPC chromatographs of polymer **3** reveal peak broadening and a shift to later retention volumes compared to the initial polymer before degradation (Fig. 1). After 4 days, the polymer peak has nearly completely disappeared suggesting a rapid decrease of the initial molecular weight. Furthermore, a second peak arises at 11.6 mL, corresponding to a molecular weight of around 1000 g mol^−1^ which can be directly assigned to the cleavage of the M-1000-Val side groups.

**Figure 2:**
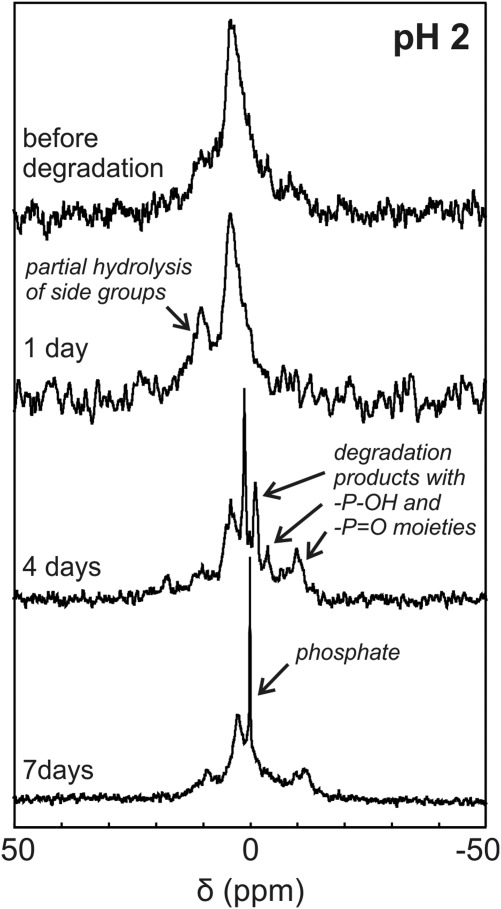
Degradation studies of polymer 3 with ^31^P NMR spectroscopy under accelerated degradation conditions in acidified D_2_O (pH 2) over 7 days. Arising signals can be associated with the formation of various intermediates with =P=OH and =P=O moieties, as well as breakdown of the polyphosphazene backbone. The peak for inorganic phosphate can be observed at around 0 ppm.

The breakdown of the polymers was further studied with ^31^P NMR spectroscopy to monitor intermediate polyphosphazene degradation products. To illustrate this, the changes for polymer **3** observed in the ^31^P NMR spectra during the hydrolysis, also under enhanced degradation conditions at pH 2, are depicted in Figure 2. As expected, the degradation proceeded rapidly at pH 2 (see later section on pH effect), as indicated by the appearance of several peaks after 4 days. These additional ^31^P resonances at −1.1 ppm, −3.8 ppm, and −9.8 ppm stem from the formation of =P=OH, =P=O, and products of geminal hydrolysis.^28^,^41^ After 7 days, the sharp peak at around 0 ppm dominated the spectrum, which is associated with the formation of inorganic phosphate. These results support the proposed degradation mechanism of polyphosphazenes that involves the hydrolysis and thus hydrolytic cleavage of the side groups from the phosphazene backbone, leading to hydroxyphosphazenes and phosphazanes.^31^

#### Spacer Effect

**Figure 3:**
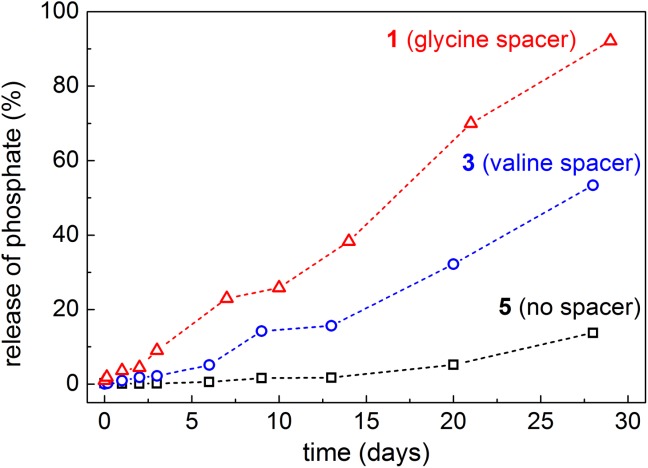
Degradation profiles of polymers 1 (Δ), 3 (○), and 5 (□) at pH 5. The increasing amount of phosphate was quantitatively determined by UV–Vis analysis. [Color figure can be viewed in the online issue, which is available at http://wileyonlinelibrary.com.]

Various degradation pathways have been proposed, all of which result in hydrolytic chain cleavage yielding low molecular weight fragments up to the formation of phosphates and ammonia.^31^ For this reason, the increase in phosphate formation, a final backbone degradation product, was measured (pH 5, 37 °C) for polymers **1, 3**, and **5** (Fig. 3). Polymer **5**, without an amino acid spacer, degraded at the slowest rate, with only 14% of the backbone degrading to phosphate after 4 weeks. However, the incorporation of an amino acid spacer was observed to have a considerable destabilization effect, with polymer **1**, incorporating the glycine spacer, degrading almost completely within 4 weeks, and thus hydrolyzing significantly faster than polymer **5**. With the valine spacer (polymer **3**), the phosphate formation was retarded in comparison to polymer **1** (glycine spacer) but faster than polymer **5** (no spacer), with half of the polyphosphazene backbone being degraded into phosphate after 4 weeks. The hydrolytic stabilities thus decreased from polymer **5** > **3** > **1**.

The incorporation of amino acid ester units is of particular importance for the synthesis of hydrolytically sensitive polyphosphazenes,^21^,^42^ with a proposed hydrolysis of the side group ester linkage leading to the formation of carboxylic acids that promote backbone cleavage and thus enhance the hydrolysis of the polyphosphazene backbone.^17^,^43^ However, in this work, the hydrophilic side chains are grafted via amide bonds onto the amino acid spacer. For this reason, it would seem reasonable to exclude, as previously proposed,^31^ hydrolysis of the ester groups on the degradation behavior of the polymers. As amide degradation is not to be expected, it would appear, therefore, that the adjacent carbonyl groups from the amino acid spacer play a significant role in accelerating the degradation rate of the polyphosphazene backbone, perhaps via a nucleophilic attack from the carbonyl oxygen onto the phosphorus atoms in the polyphosphazene backbone.^21^ The observation that polymer **3**, with a valine spacer, degrades slower than polymer **1**, with a glycine spacer, is presumed to be due to shielding of the backbone from hydrolytic attack from the isopropyl groups at the alpha-C-atoms and corresponds well with previous work involving amino acid ester substitutents.^22^

The hydrolysis process was further monitored by ^31^P NMR spectroscopy under neutral conditions over 9 weeks (Supporting Information Fig. SI-5) with the trend corresponding well to that observed for the phosphate testing. Polymer **1** is observed to degrade rapidly, whereas, polymer **2**, with 50% of the glycine-functionalized M-1000 and 50% of the M-1000 added, showed a significantly higher aqueous stability. Thus addition of M-1000, with no spacer, has a considerable stabilizing effect on the glycine polymer. However, although with such a mixed macromolecular substitution approach is a simple route to reduce the hydrolytic sensitivity of these polyphosphazenes, there is a distinct lack of control and thus reproducibility associated with the ratio of side-groups attached via a mixed substituent method and even when a step-wise synthesis is performed, exchange^44^ at the phosphorus atom is inherently possible. For this reason, it was decided to concentrate these studies on polymers **1, 3**, and **5**, where stoichiometric control can be guaranteed.

#### pH Effect

**Figure 4:**
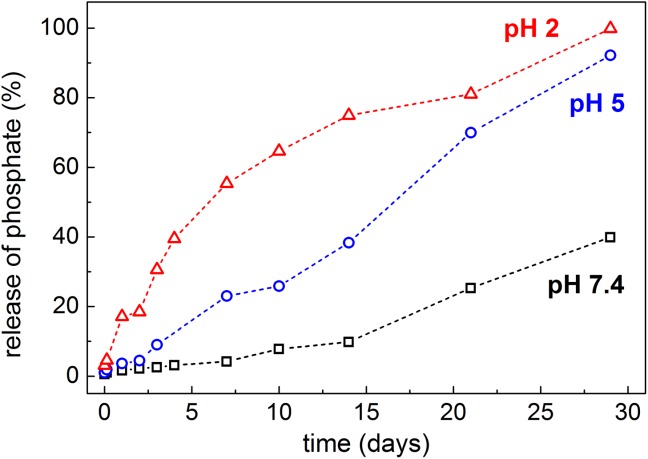
Figure Release of inorganic phosphate of polymer 1 under aqueous conditions at pH 2 (Δ), pH 5 (○), and pH 7.4 (□), showing the enhanced degradation at reduced pH, as measured by UV–Vis spectroscopy. [Color figure can be viewed in the online issue, which is available at http://wileyonlinelibrary.com.]

The influence of the pH-value on the degradation profile was also investigated. The degradation profiles of polymer **1** under acidic and neutral conditions (Fig. 4) revealed the promoting influence of the pH-value on the degradation process, with a slowly increasing formation of phosphate at pH 7 and considerably higher degradation rates observed at pH 5 and 2 for all polymers. Under neutral conditions only 40% of the polyphosphazene backbone was hydrolyzed within 4 weeks. In mildly acidic environment, however, a continuous hydrolytic chain cleavage and thus increase of phosphate could be detected, with 90% of polymer **1** being degraded after 4 weeks. At pH 2, however, a significantly faster initial degradation rate was observed, with 50% of the backbone being degraded after 6 days and complete degradation after 4 weeks.

Polymer **1** with the glycine spacer degraded completely within 4 weeks at pH 2, the degradation of the relatively stable polymer **5** was also significantly faster under strong acidic conditions (Supporting Information Fig. SI-6). Thus, the degradation of all studied polymers proceeded substantially faster as the pH-value was decreased, supporting the findings of the degradation studies using UV–Vis spectroscopy to investigate the formation of phosphate. A similar accelerated degradation behavior at lower pH-values has also been observed for polyphosphazenes containing *N*-ethylpyrrolidone polyphosphazene substituents^28^ as well as for phosphoramidate forming polyphosphoesters.^45^

These results would appear to confirm the proposed mechanism that a protonation of the polyphosphazene backbone in an acidic environment increases the overall hydrolysis rate of the polymers.^15^,^46^–^48^ Subsequent cleavage of the side chains leads to an exposure of the backbone that is then more accessible to hydrolytic attack.

### Cell Viability Studies

**Figure 5:**
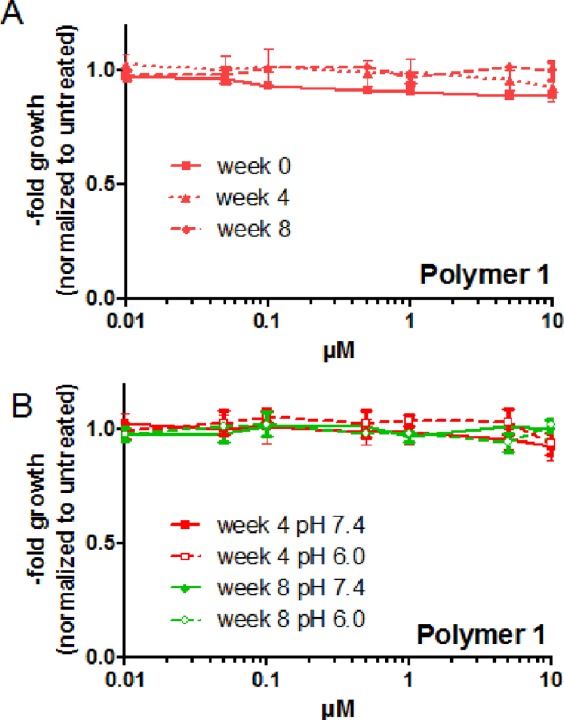
Cell viability of HCT116 cells after incubation with polymer 1. (A) Activity of HCT116 cells was tested by MTT assay after 72 h incubation. Normalized values given are mean values ± S.D. of experiments performed in triplicate. (B) Comparison of standard cell culture conditions (pH 7.4) and mildly acidic milieu (pH 6, MES buffer) on the biocompatibility of polymer 1. [Color figure can be viewed in the online issue, which is available at http://wileyonlinelibrary.com.]

A selection of the polymers underwent preliminary testing for their biocompatibility, in particular for potential use as macromolecular drug carriers. For this purpose, polymer **1** (glycine spacer), polymer **3** (valine spacer), and polymer **5** (no spacer) were tested with HCT116 cells. As shown in Figure 5(A) and Supporting Information Figure SI-7A, none of the polymers showed a substantial impact on cell viability. Furthermore, the partially degraded polymer (stored for 4 or 8 weeks at 37 °C in aqueous solution) did not show any harmful effect on the cell viability. To mimic acidic degradation conditions and thus possible intermediates, additional experiments were performed in MES-buffered medium at pH 6.0 [Fig. 5(B) and Supporting Information Fig. SI-7B]. Also in these tests, no substantial impact on the cell viability was observed compared to controls. Similar results were also observed in A2780 and Hep3B cells (data not shown) confirming the non-toxic nature of these polymers and degradation products within a polymer concentration range relevant for drug delivery applications (0.01–10 μM).

## Conclusions

A series of novel graft poly(organophosphazenes) were synthesized containing hydrophilic Jeffamine M-1000 side chains that were coupled via an amino acid spacer onto the degradable polyphosphazene backbone. The polymers showed excellent solubility in water and provide a pH-assisted degradation behavior with a considerably faster degradation at lower pH-values. The hydrolytic stability was tailored by careful choice of the amino acid spacer and could be easily increased by steric shielding of the polymer backbone via the R groups of the alpha-C-atom of the amino acid spacer. The biocompatibility demonstrated by these water soluble polymers, their degradation intermediates and products, in combination with their synthetic structural control and broad spectrum of degradation rates available suggest significant promise as materials for aqueous biomedical applications such as polymer therapeutics.
