# Supplementary material for: Water-Soluble, Biocompatible Polyphosphazenes with Controllable and pH-Promoted Degradation Behavior
Source: J Polym Sci A Polym Chem. 2013 Nov 22;52(2):287–94. doi: 10.1002/pola.27002 (PMC3980369; doi:10.1002/pola.27002)
Supplement: Supplementary file 1 [file pola0052-0287-sd1.pdf]

Supporting Information

Characterisation of precursors.

M-1000-Glycine. Yield 4.84 g (84 %). ^{1}H NMR (300 MHz, $CDCl_3$): δ 1.07 (m, 8H), 1.37 (s, 9H), 3.30 (s, 3H), 3.57 (s, 87H) ppm.

M-1000-Valine. Yield 6.39 g (71 %). ^{1}H NMR (300 MHz, $CDCl_3$): δ 0.90 (m, 6H), 1.10 (m, 8H), 1.40 (s, 9H), 2.60 (br, 1H), 3.34 (s, 3H), 3.60 (s, 80H), 4.08 (br, 1H) ppm.

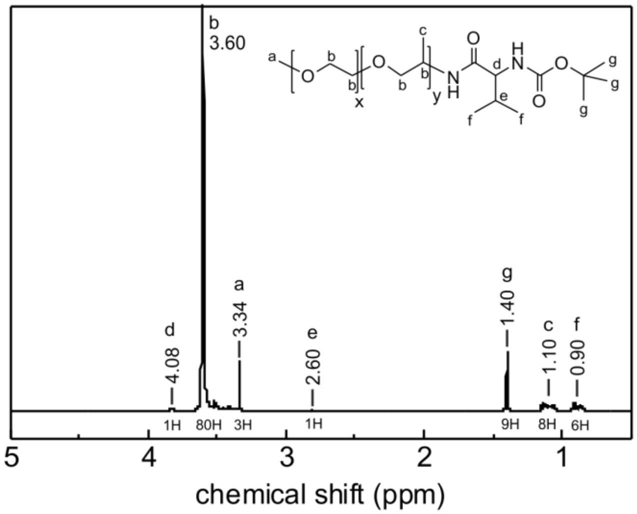

Figure SI-1. ^{1}H NMR spectrum of M-1000-Valine in $CDCl_3$.

Characterisation of polymers 1 - 5.

Polymer **1**. Yield 0.43 g (45 %). FTIR (solid): ν_{max} = 2883 (C-H), 1663 (C=O), 1103 (C-O) cm^{-1}, ^{1}H NMR (500 MHz, D_2O, δ): 0.98 (br, 7H), 3.20 (s, 3H), 3.52 (br, 80H) ppm. ^{31}P NMR (202 MHz, D_2O, δ): -8.04 - 13.23 (very broad) ppm. GPC: M_n = 20 178 g mol-1, M_w 23 380 = g mol-1, M_w / M_n = 1.16.

Polymer **2**. Yield 0.45 g (32 %). FTIR (solid): ν_{max} = 2883 (C-H), 1660 (C=O), 1108 (C-O) cm^{-1}, ^{1}H NMR (500 MHz, D_2O, δ): 0.99 (m, 8H), 3.21 (s, 3H), 3.53 (s, 83H) ppm. ^{31}P NMR (202 MHz, D_2O, δ): 3.41 ppm. GPC: M_n = 11 430 g mol^{-1}, M_w = 15 314 g mol^{-1}, M_w / M_n = 1.34.

Polymer **3**. Yield 0.57 g (38 %). FTIR (solid): ν_{max} = 2883 (C-H), 1653 (C=O), 1109 (C-O) cm^{-1}, ^{1}H NMR (500 MHz, D_2O, δ): 0.99 (m, 9H), 3.21 (s, 3H), 3.53 (s, 82H) ppm. ^{31}P NMR (202 MHz, D_2O, δ): 1.05 ppm. GPC: M_n = 13 296 g mol-1, M_w = 17 849 g mol-1, M_w / M_n = 1.34.

Polymer **4**. Yield 0.63 g (66 %). FTIR (solid): ν_{max} 2883 = (C-H), 1663 (C=O), 1107 (C-O) cm^{-1}, ^{1}H NMR (500 MHz, D_2O, δ): 0.98 (br, 8H), 3.20 (s, 3H), 3.52 (80H) ppm. ^{31}P NMR (202 MHz, D_2O, δ): 1.53 ppm. GPC: M_n = 15 101 g mol^{-1}, M_w = 18 143 g mol^{-1}, M_w / M_n = 1.20.

Polymer **5**. Yield 1.21 g (67 %). FTIR (solid): ν_{max} = 3305 (N-H), 2884 (C-H), 1109 (C-O) cm^{-1}, ^{1}H NMR (300 MHz, $CDCl_3$, δ): 1.11 (m, 6H), 3.36 (s, 3H), 3.63 (2, 61H) ppm. ^{31}P NMR (202 MHz, D_2O, δ): 1.02 ppm. GPC: M_n = 14 348 g mol^{-1}, M_w = 17 709 g mol^{-1}, M_w / M_n = 1.23.

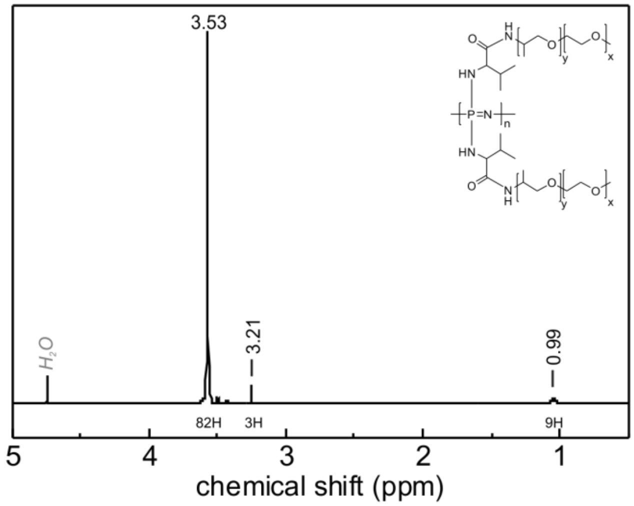

Figure SI-2. ^{1}H NMR spectrum of polymer **3** in D_2O. The signals of the valine spacer are overlaid by the large Jeffamine M-1000 signals.

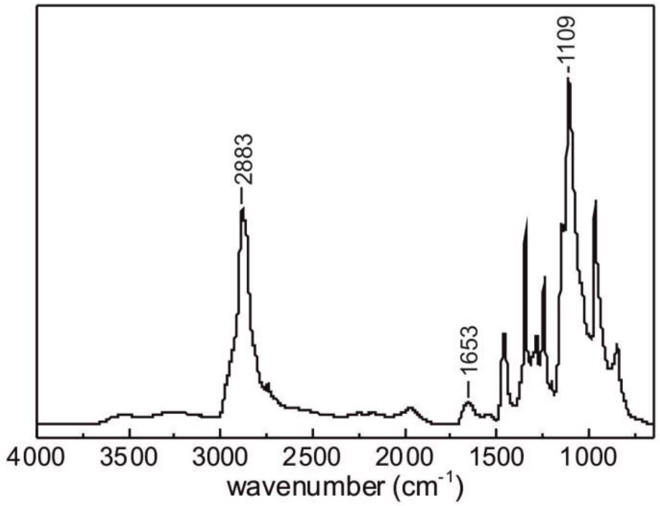

Figure SI-3. ATR-FTIR spectrum of polymer **3**. Significant bands include the C=O band at 1653 cm^{-1} stemming from the valine spacer, a large C-H band at 2883 cm^{-1} and a C-O band at 1109 cm^{-1} resulting from the M-1000 side chains.

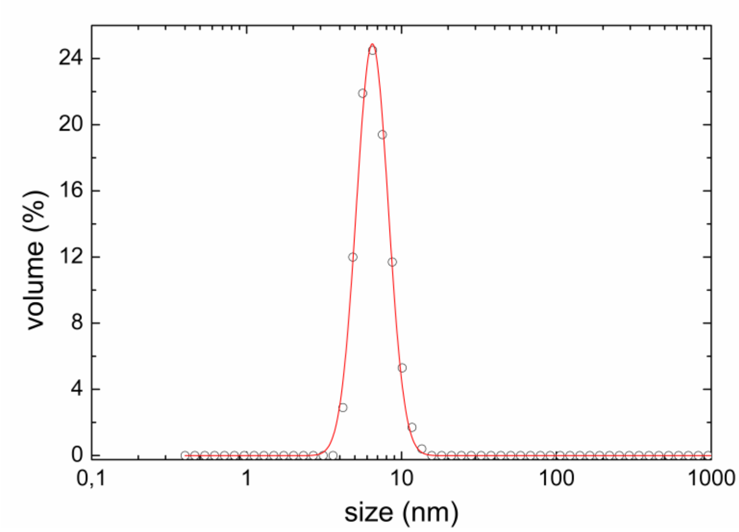

Figure SI-4. Volume size distribution of polymer **3** in deionized H_2O with a concentration of 1 mg ml^{-1} at 25°C. The mean value of the hydrodynamic diameter was calculated from the fitted curve (7.0 nm).

Degradation studies using ^{31}P NMR spectroscopy.

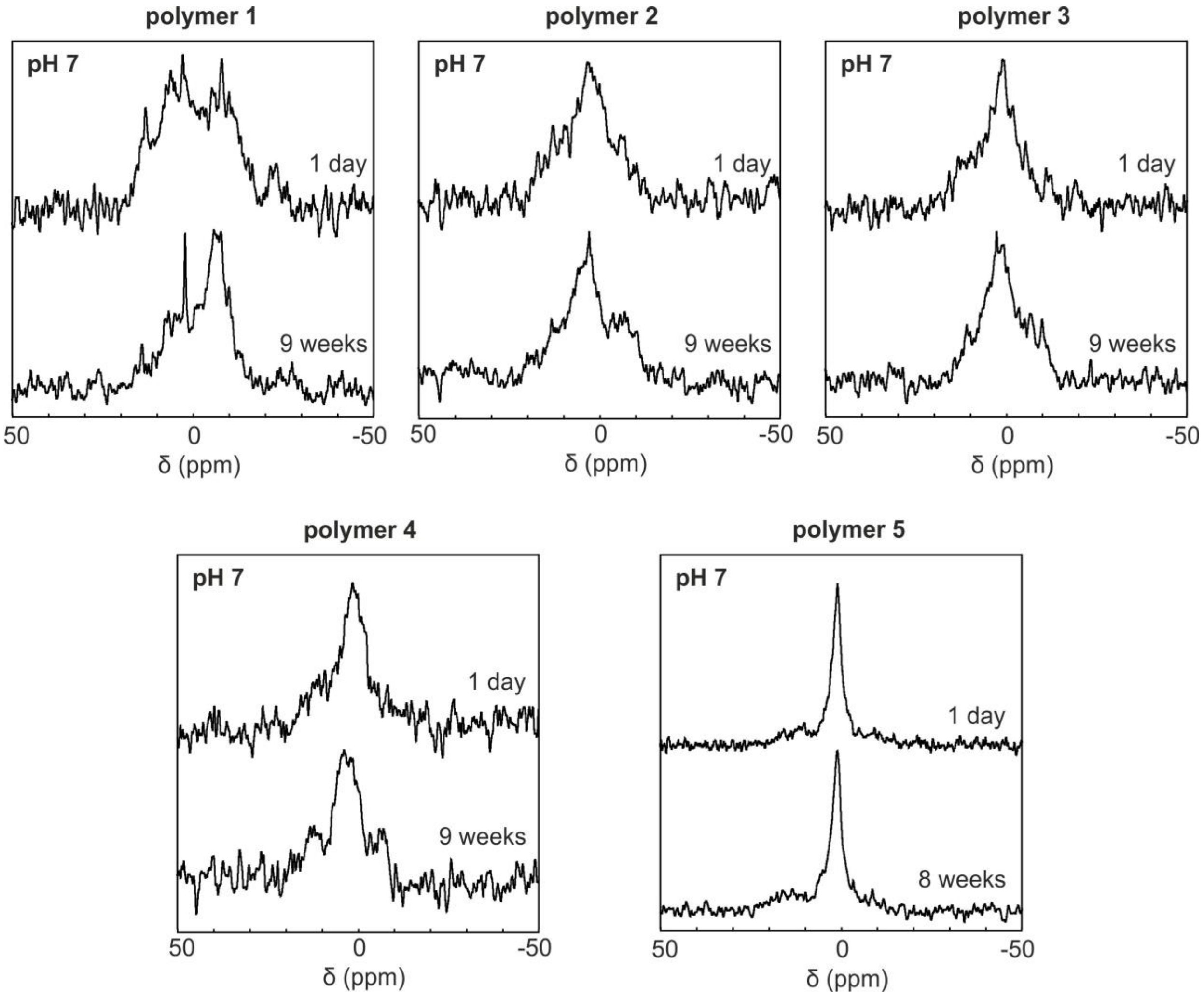

Figure SI-5. Degradation studies with ^{31}P NMR spectroscopy in D_2O (pH 7) indicating a relatively high stability of polymers **2**-**5** over 2 months. Changes in the phosphorus peak can be observed, for the glycine polymer **1**, therefore degrading significantly faster than polymers **2**-**5**. The rate of degradation of polymer **1** could be decreased by a mixed substitution (polymer **2**), i.e. 50 % of the side chains were substituted by the amino acid spacer glycine and 50 % were substituted with M-1000.

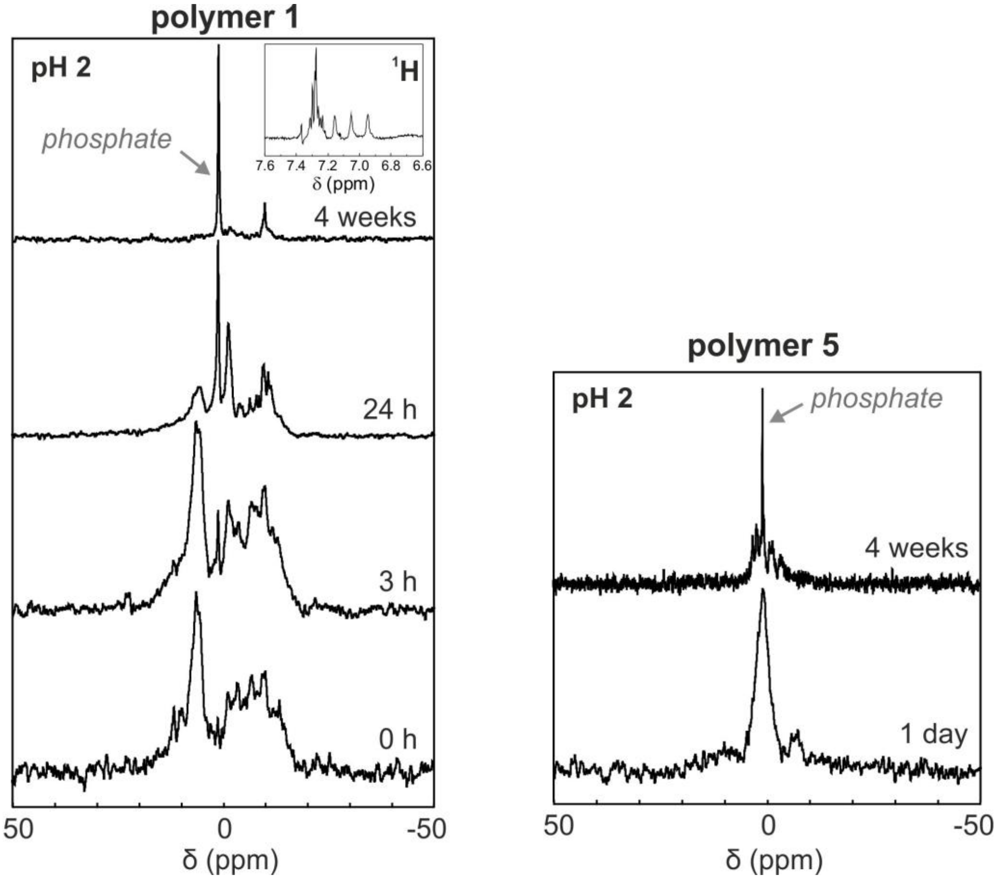

Figure SI-6. Degradation studies of polymer **1** and **5** with ^{31}P NMR spectroscopy in acidified D_2O (pH 2) over 4 weeks. One main phosphoric component resulting in a sharp signal at 1.2 ppm could be observed after four weeks indicating complete degradation of the polyphosphazene backbone into phosphate. The ^{1}H NMR spectrum of the sample after four weeks showed weak signals in the range of 6.9 to 7.2 ppm with a typical splitting, confirming the presence of ammonium, the second degradation product of the polyphosphazene backbone. Degradation studies of polymer **5** confirmed the presence of a considerable amount of phosphate (1.1 ppm) and thus accelerated degradability under harsh acidic conditions.

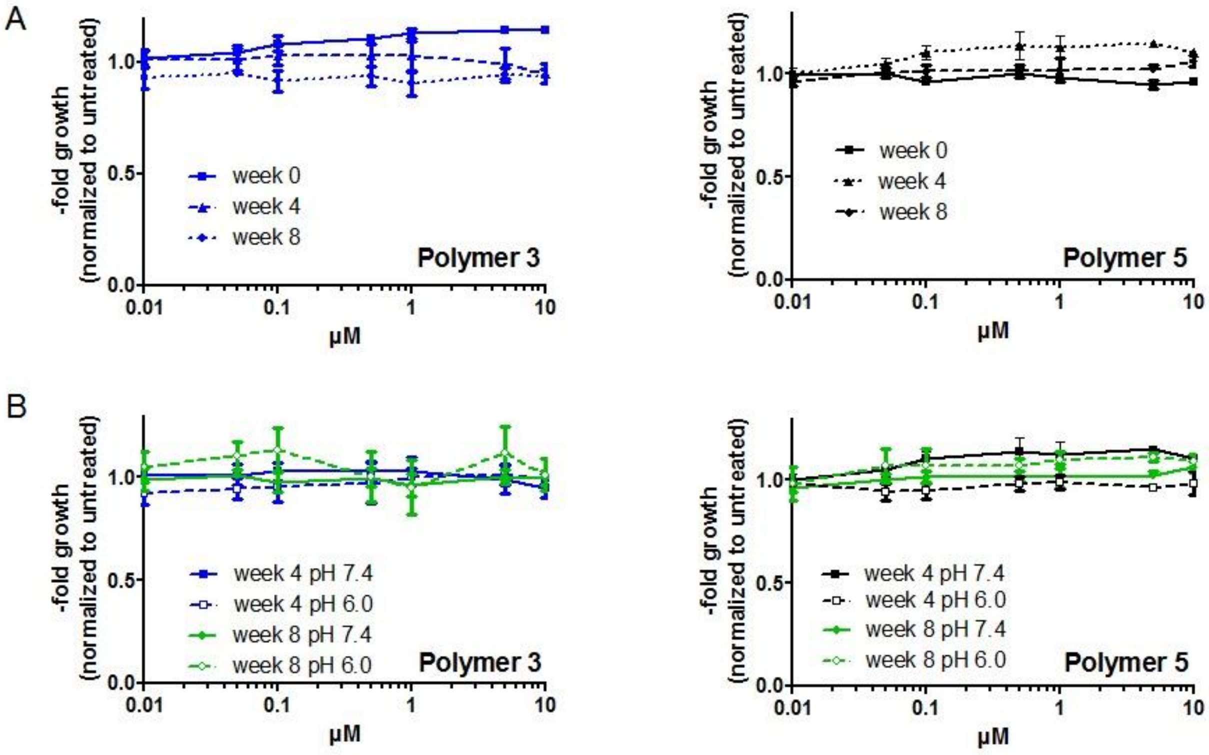

Figure SI-7. Cell viability of HCT116 cells after incubation with (A) polymer **3** (left panel) and polymer **5** (right panel), tested by MTT assay after 72 h incubation. Normalized values given are mean values ± S.D. of experiments performed in triplicate. (B) Comparison of standard cell culture conditions (pH 7.4) and mildly acidic milieu (pH 6.0, MES buffer) of polymer **3** (left panel) and polymer **5** (right panel) confirming the nontoxic nature of the polymers and their degradation products over eight weeks.
